# Letters to a Young Physician

**Published:** 1855-12

**Authors:** 


Art. IX.—*Letters to a Young Physician.* By James Jackson, M. D., Professor Emeritus of Physic in the University of Cambridge, etc., etc. Boston: 8vo. pp. 344. 1855.

The oldest physician cannot help being pleased and the youngest delightfully instructed while perusing this admirably written volume. We have read it with delight, and so must every one into whose hands it may fall. D. W. Y.

